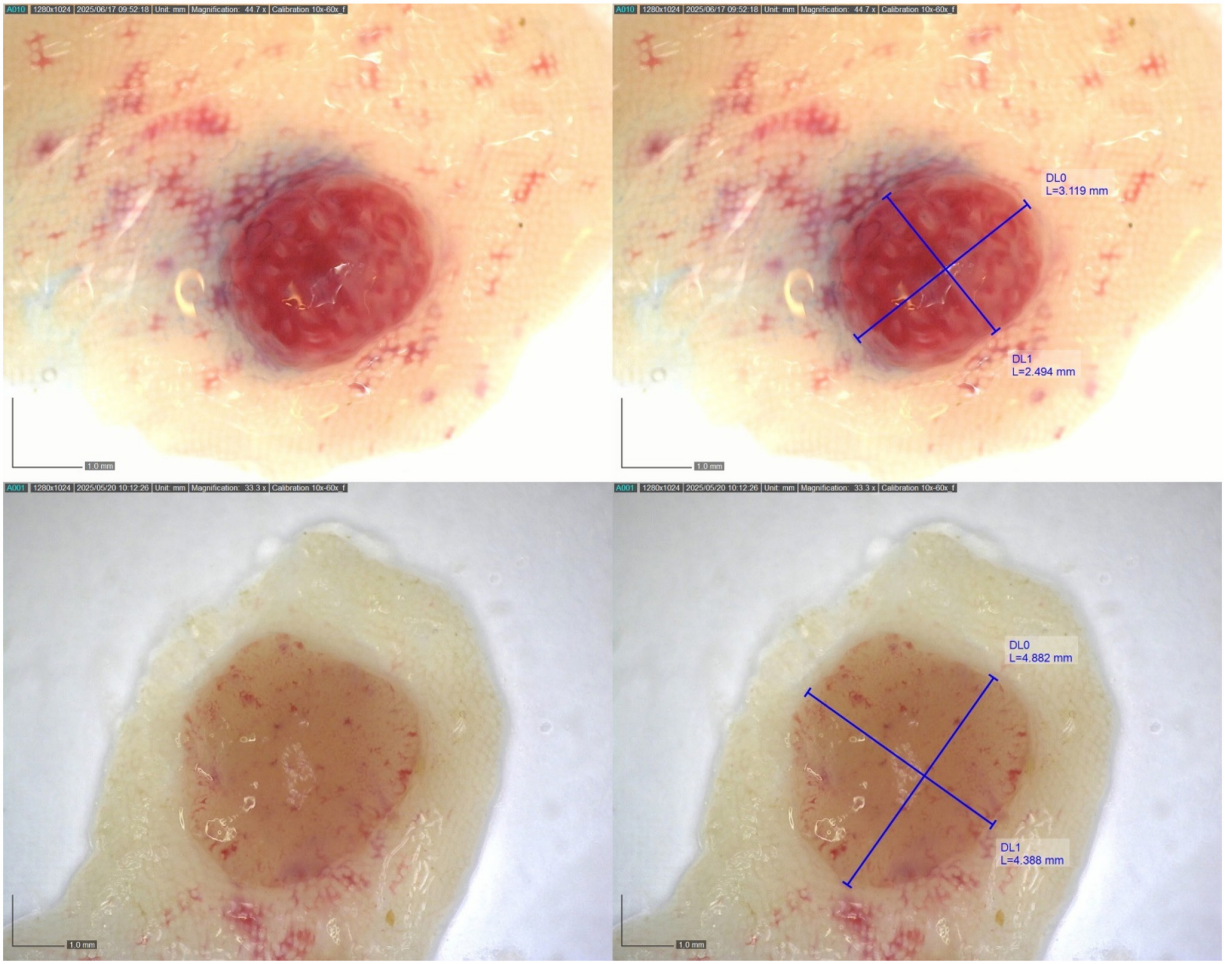# Poster Session I - A48 DIGITAL MICROSCOPY IN THE ENDOSCOPY SUITE: A REFERENCE STANDARD FOR COLORECTAL POLYP SIZE MEASUREMENT

**DOI:** 10.1093/jcag/gwaf042.048

**Published:** 2026-02-13

**Authors:** P Aleksieva, R Djinbachian, D K Rex, H Pohl, N Shahidi, S Mitchell, M Mahdadi, É Cristea, M Oleksiw, V Michal, C Gefflot, D von Renteln

**Affiliations:** Centre Hospitalier de l’Universite de Montreal, Montreal, QC, Canada; Centre Hospitalier de l’Universite de Montreal, Montreal, QC, Canada; Indiana University School of Medicine, Indianapolis, IN; Dartmouth College Geisel School of Medicine, Hanover, NH; The University of British Columbia Faculty of Medicine, Vancouver, BC, Canada; Centre Hospitalier de l’Universite de Montreal, Montreal, QC, Canada; Centre Hospitalier de l’Universite de Montreal, Montreal, QC, Canada; Centre Hospitalier de l’Universite de Montreal, Montreal, QC, Canada; Centre Hospitalier de l’Universite de Montreal, Montreal, QC, Canada; Centre Hospitalier de l’Universite de Montreal, Montreal, QC, Canada; Centre Hospitalier de l’Universite de Montreal, Montreal, QC, Canada; Centre Hospitalier de l’Universite de Montreal, Montreal, QC, Canada

## Abstract

**Background:**

Accurate determination of colorectal polyp size is critical for surveillance recommendations and for generating reliable ground truth in artificial intelligence (AI) development. Visual estimation during endoscopy is imprecise, and no validated reference standard exists.

**Aims:**

We aimed to assess the accuracy and reproducibility of real-time digital microscopic measurement of fresh polypectomy specimens.

**Methods:**

In this prospective study at the Centre hospitalier de l’Université de Montréal (CHUM), 70 polyps from 44 patients (mean age 65.4 years; 52.3% female) were measured on-site using a calibrated digital microscope. Three independent raters, blinded to each other, obtained long- and short-axis measurements. The primary outcome was inter-rater reliability for long-axis measurements. Secondary outcomes included short-axis reliability, overall agreement, and classification (≤5 mm vs. >5 mm).

**Results:**

Of the included lesions, 65.7% were adenomas, 14.3% hyperplastic, and 10.0% inflammatory. Inter-rater reliability was excellent for both long-axis (ICC 0.941, 95% CI 0.914–0.961) and short-axis (ICC 0.943, 95% CI 0.917–0.962) measurements. Bland–Altman plots demonstrated mean differences close to zero across all rater pairs, without systematic bias. Limits of agreement were within ±1 mm. When dichotomized at the 5 mm threshold, agreement was perfect (κ = 1.0).

**Conclusions:**

On-site digital microscopic measurement of fresh polyps provides highly reproducible, unbiased, and verifiable size data with photographic documentation. This method should be considered the reference standard for establishing accurate ground truth datasets in the development and validation of AI-assisted polyp sizing models.

A47 Table 1: Polyp characteristics

**Funding Agencies:**

None